# Expression and activity of eIF6 trigger Malignant Pleural Mesothelioma growth *in vivo*

**DOI:** 10.18632/oncotarget.5462

**Published:** 2015-10-06

**Authors:** Annarita Miluzio, Stefania Oliveto, Elisa Pesce, Luciano Mutti, Bruno Murer, Stefano Grosso, Sara Ricciardi, Daniela Brina, Stefano Biffo

**Affiliations:** ^1^ Molecular Histology and Cell Growth Unit, Istituto Nazionale Genetica Molecolare, “Romeo ed Enrica Invernizzi”, Milano, Italy; ^2^ Dipartimento di Scienze e Innovazione Tecnologica, University of Eastern Piedmont, Alessandria, Italy; ^3^ Biomedicine Institute, The University of Salford, The Crescent, Salford, UK; ^4^ Hospital Dall'Angelo, Pathology Unit, Venice, Italy; ^5^ Medical Research Council Toxicology Unit, Leicester, UK; ^6^ Department of Biosciences, University of Milan, Milan, Italy

**Keywords:** Malignant Pleural Mesothelioma, eIF6 phosphorylation, anti-association activity, PKCbeta, Enzastaurin

## Abstract

eIF6 is an antiassociation factor that regulates the availability of active 80S. Its activation is driven by the RACK1/PKCβ axis, in a mTORc1 independent manner. We previously described that eIF6 haploinsufficiency causes a striking survival in the Eμ-Myc mouse lymphoma model, with lifespans extended up to 18 months. Here we screen for eIF6 expression in human cancers. We show that Malignant Pleural Mesothelioma tumors (MPM) and a MPM cell line (REN cells) contain high levels of hyperphosphorylated eIF6. Enzastaurin is a PKC beta inhibitor used in clinical trials. We prove that Enzastaurin treatment decreases eIF6 phosphorylation rate, but not eIF6 protein stability. The growth of REN, *in vivo*, and metastasis are reduced by either Enzastaurin treatment or eIF6 shRNA. Molecular analysis reveals that eIF6 manipulation affects the metabolic status of malignant mesothelioma cells. Less glycolysis and less ATP content are evident in REN cells depleted for eIF6 or treated with Enzastaurin (Anti-Warburg effect). We propose that eIF6 is necessary for malignant mesothelioma growth, *in vivo*, and can be targeted by kinase inhibitors.

## INTRODUCTION

Protein synthesis (translation) is essential for cell growth. It is regulated by ribosome synthesis in the nucleolus and ribosome usage in the cytoplasm. Translation is deregulated in cancer cells [1, 2]. Recent works have shown that the translational machinery plays an active role in transformation and tumor malignancy, suggesting that it can be a therapeutic target [3, 4]. Furthermore, mutations of ribosome-associated factors have been described in sporadic cancer, i.e. rpL5 and rpL10 in T-cell acute lymphoblastic leukemia [5], stressing the naivety of the simple correlation between growth and ribosomal biology.

In eukaryotic cells, the initiation phase of translation is rate-limiting for a given mRNA. Throughout initiation, Initiation Factors (eIFs) act as regulators, downstream of signalling events [6]. Briefly, initiation requires three consecutive steps: formation of 43S pre-initiation complex through the recruitment of the ternary complex eIF2-GTP-tRNAi(met) on the small 40S ribosomal subunit; formation of 48S by binding of mRNA in complex with eIF4F to 43S; recruitment of large 60S subunits and formation of elongating 80S. The best-characterized oncogenic signalling acting on initiation is driven by the PI3K-AKT-mTOR pathway that ultimately regulates eIF4F activation and 48S formation [7]. eIF4F comprises the cap-binding protein eIF4E, the scaffold protein eIF4G and the ATP-dependent helicase eIF4A. eIF4E activity is inhibited by dephosphorylated 4E-BPs binding proteins. Biochemical, genetic and pharmacological evidences show that either inhibition of 4E-BP activity [8, 9] or activation of eIF4E [10, 11] leads to cancer progression. mTORc1 complex phosphorylates 4E-BPs. Rapamycin and its clinical derivatives (rapalogs) such as everolimus are inhibitors of mTORc1 complex. Rapalogs impair eIF4F formation and block the growth of sensitive tumors. In several contexts, the sensitivity to rapalogs depends from the ratio of eIF4E/4E-BPs. Increase in the eIF4E/4E-BP ratio attenuates the mTORc1 inhibition and viceversa [12–14]. Rapamycin sensitivity can also be independent from eIF4E/4E-BP ratio, due to multiple mTORc1 substrates [15]. Moreover, the cytostatic effect of rapamycin is transient. It was reported that, in different tumoral cell lines, 4E-BP1 phosphorylation re-emerges after short-term treatment of rapamycin, despite continued inhibition of S6K, resulting in a recovery of cap-dependent translation [16]. Importantly, the drug is highly effective in the presence of PI3K mutations [17]. In contrast, cancer cells with mutations in the RAS pathway are resistant to mTORc1 inhibition [17], demonstrating the existence of either alternative initiation factors or pathways converging on translation which control tumor growth.

eIF6 is an initiation factor driven by RACK1-PKCβII axis, independently from mTORc1. Mechanistically, eIF6 binds 60S ribosomal subunits and has an anti-association property, by impeding 60S premature joining to 40S [18]. Although mostly cytoplasmic, a minor pool of eIF6 is essential for nucleolar maturation of 60S subunits [19]. In general, eIF6 is rate limiting for tumor onset and progression. eIF6 haploinsufficient cells are normal, but not efficiently transformed *in vitro* [20]. In a mouse model of Myc-driven lymphomagenesis, eIF6 heterozygous mice survive much longer, even more than one year, when compared to the 4-months life expectancy of wt mice [21]. eIF6 phosphorylation of Ser235 has been reported in several tumor cells [22]. PKCβII kinase is recruited by the scaffold protein RACK1, leading to eIF6 phosphorylation on Ser235, allowing eIF6 activation [23, 24]. RACK1/PKC expression confers chemoresistance [25]. Consistently, transformed fibroblasts with eIF6^S235A^ show resistance to oncogenic transformation and reduced growth *in vivo* [21]. In human cancers, eIF6 is highly expressed in colorectal carcinomas, and its overexpression is associated with tumor stage [26]. Recently, eIF6 has been identified as one of 21 essential genes amplified in highly proliferative luminal-subtype human breast cancer [27]. Open questions are, i) which tumors rely on eIF6 expression and/or activation for growth, and ii) how feasible and effective is eIF6 targeting.

Malignant pleural mesothelioma (MPM) is characterized by an indolent progression with almost 100% lethality. MPM is generally found to be resistant to conventional forms of therapy, such as pemetrexed and cisplatinum combination chemotherapy [28]. We recently showed that in malignant mesothelioma, translational control was altered and by large insensitive to rapamycin inhibition, suggesting that other initiation factors can sustain tumor growth [29]. This finding was supported by the observed ineffectiveness of rapalogs in MPM therapy [30]. Here we investigated the hypothesis that eIF6 can be critical for MPM growth. We found that eIF6 is overexpressed and hyperactivated in mesotheliomas and that inhibition of its expression or phosphorylation delays tumor progression.

## RESULTS

### eIF6 is a marker of aggressive Malignant Pleural Mesothelioma (MPM)

**Figure 1:**
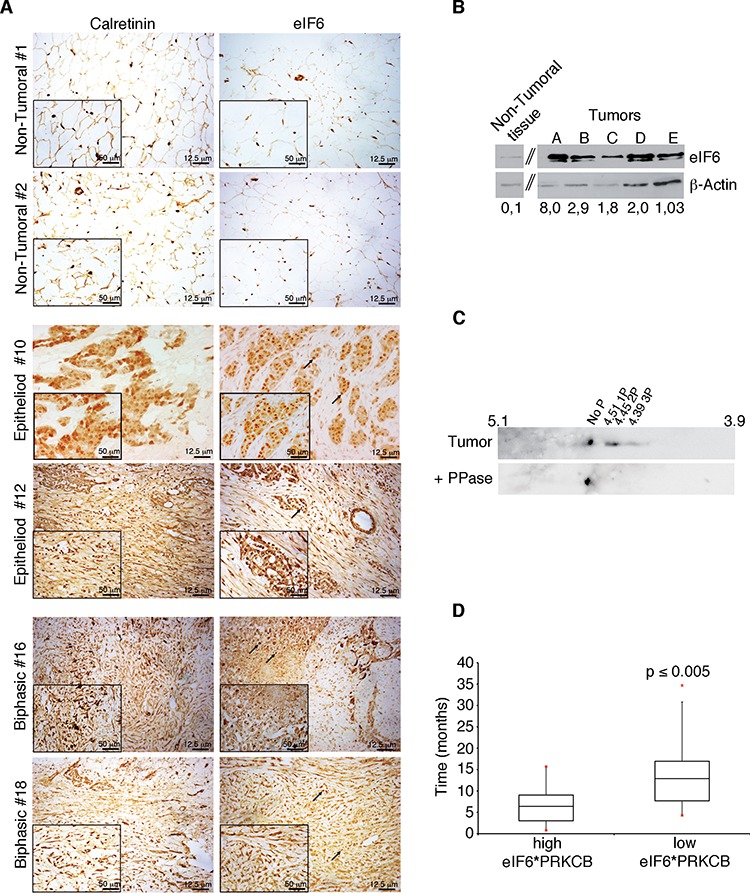
eIF6 expression and phoshorylation correlate to lower MPM patients survival **A.** IHC stainings on representative human non-tumoral samples and on biopsies of epithelial and biphasic malignant pleural mesothelioma: eIF6 expression is evident both in the nucleoli, indicated with black arrows, and in the cytoplasm of tumor cells; Calretinin is used as a positive marker of MPM tumors and scale bar is indicated. **B.** Representative Western Blot analysis of different human biopsies of malignant pleural mesothelioma: eIF6 protein levels are higher in tumor samples compared to non tumoral ones. eIF6/β-Actin Ratio is quantified by densitometric analysis, as indicated. **C.** 2-D analysis on a pool of three tumor extracts: focused spots are indicated. Treatment with PPase is used as negative control. **D.** Data mining studies reveal that high co-expression of eIF6 and PKCβ is associated to lower survival of MPM patients. Statistical analysis was performed by a paired *t*-test. *p*-value ≤ 0.005.

To study whether eIF6 protein was expressed in malignant pleural mesothelioma (MPM), we performed an immunohistochemistry staining on 24 human MPM samples from an Italian cohort, using an anti-eIF6 polyclonal antibody. Of these, 19 were epithelial, 3 sarcomatous, and 2 biphasic. All MPM cases are summarized in Supplementary Table S1. Representative stainings of epithelioid and biphasic histotypes of MPM are shown in Figure 1A and Supplementary Figure S1. Human epithelioid biopsies showed widespread mesothelioma infiltration that presented, with different prevalence, epithelial and connective components. Tumor components were characterized by islands or tubular formations. Biphasic (mixed) histotypes showed both spindle-shaped cells, typical of sarcomatoid subtype, and epithelial areas. In all analyzed cases, eIF6 was expressed at high levels both in the nucleoli (black arrows) and in the cytoplasm of MPM cells (Figure 1A). Nucleoli were enlarged, suggesting abnormal ribosome biogenesis. By using calretinin as a diagnostic marker for MPM, we confirmed that eIF6 overexpression was limited to tumor cells. Conversely, both eIF6 and calretinin are less expressed in non-tumoral lung biopsies. (Figure 1A). Next, we evaluated both eIF6 expression and phosphorylation on human MPM epithelial tumor samples excised. These samples were from Glenfield Hospital, Leicester, UK. First, we confirmed by Western Blot analysis that eIF6 overexpression is a constitutive feature of MPM (Figure 1B). Control, non tumoral cells were from primary human mesothelium. Second, 2-D electrophoresis on a pool of three tumoral samples displayed 3 well-focused spots compatible with eIF6 phosphorylation sites. Tumors treated with phosphatase showed a single focused spot (Figure 1C).

Last, we data-mined eIF6 mRNA levels from MPM microarray studies [31]. Data showed that 35/42 MPM patient datasets expressed higher levels of eIF6 mRNA in tumor samples. However, no relationship between eIF6 mRNA levels at time of analysis and survival was observed. eIF6 can be phosphorylated by the RAS/PKC pathway [23]. We data-mined on mesothelioma datasets the expression of PKCβ (PRKCB), the favoured RACK1 partner [32]. Combined expression of PRKCB and eIF6 was then used to evaluate survival. Strikingly, high eIF6/high PRKCB expression correlated with lower survival, *p* ≤ 0.005 (Figure 1D). In conclusion, analysis of three separate mesothelioma datasets showed that the combination of eIF6 expression and phosphorylation correlates with negative survival, raising the question whether its inhibition may be beneficial.

### eIF6 hyperphosphorylation in MPM cell line REN

**Figure 2:**
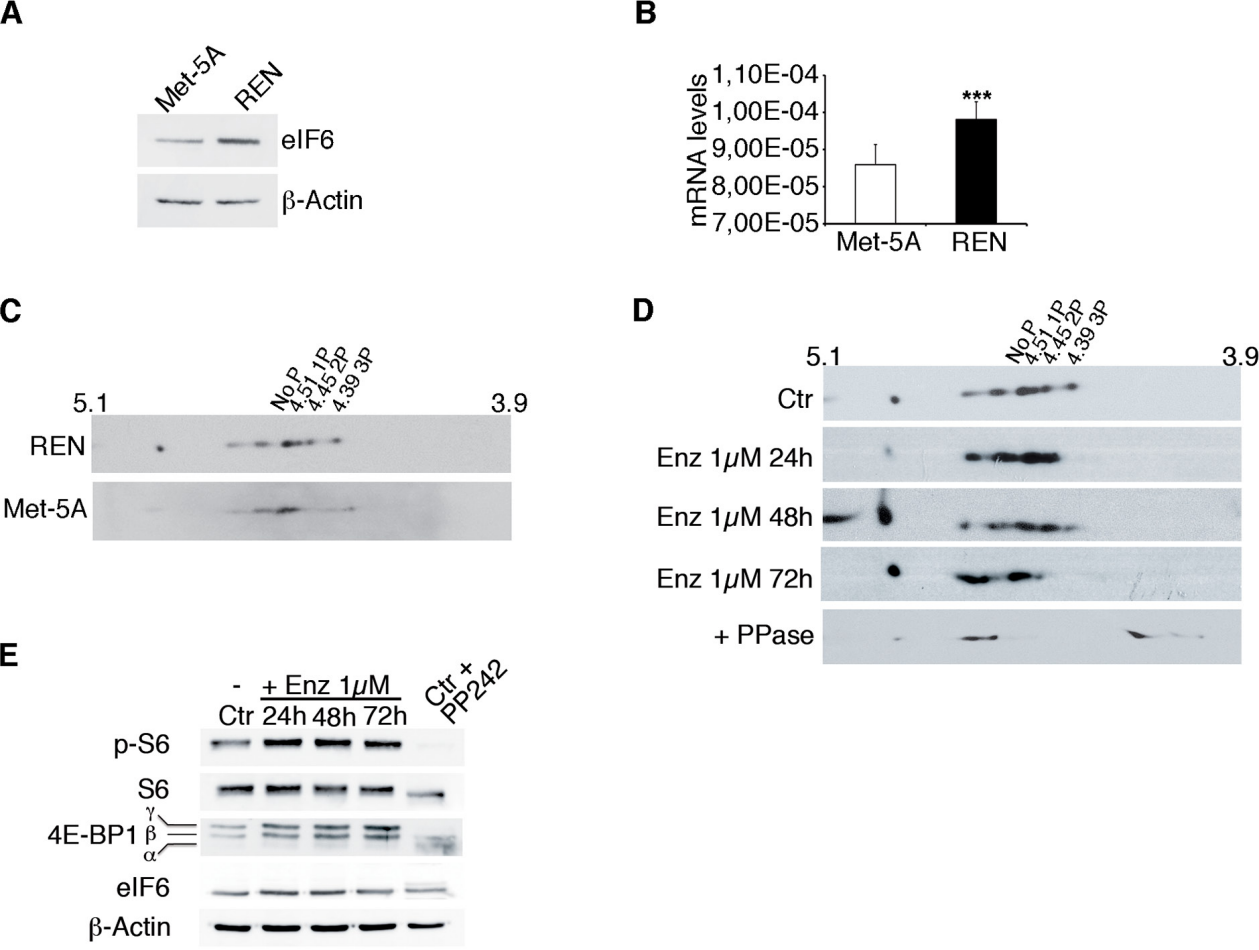
eIF6 hyperphosphorylation in REN cells is sensitive to Enzastaurin treatment **A.** Western Blot analysis shows that eIF6 is overexpressed in REN cells compared to non tumorigenic Met-5A cells. **B.** Real-Time PCR confirms that eIF6 mRNA levels are increased in REN cells. **C.** Representative 2-D gel electrophoresis on REN and Met-5A cells: focused spots indicate that eIF6 is hypershosphorylated in REN cells, but not in non tumorigenic Met-5A cells. **D.** Representative 2-D gel electrophoresis on REN cells treated with 1 μM Enzastaurin for 24, 48 and 72 hours: eIF6 phosphorylation is sensitive to Enzastaurin treatment, in a time dependent manner. Lambda PPase is used as positive control of unphosphorylation state. **E.** Representative Western Blot analysis on REN cells treated with Enzastaurin at different time points indicates that mTORc1 kinase is activated: phosphorylation of rpS6 and 4E-BP1 are equivalent in control cells and upon drug treatment. PP242 treatment is used as control of mTORc1 inactivation. Data are normalized to β-Actin. All values represent the mean ± SD. Results are representative of three independent experiments. Asterisks indicate a statistically significant change obtained by two-tailed *t*-test (***p ≤ 0.001).

We analyzed the expression and phosphorylation of eIF6 in the epithelial MPM cell line, REN, and compared it to the expression of eIF6 in non-tumorigenic Met-5A mesothelial cells. We observed augmented eIF6 expression and phosphorylation in REN cells (Figure 2A, 2B, 2C). Phosphorylation of eIF6 occurs downstream of RACK1/PKC activation. PKCβ is the preferential partner of RACK1 [23]. Enzastaurin is a specific PKCβ inhibitor that has been used in clinical trials for treating B-cell malignancies, i.e. [33]. Enzastaurin (1 μM) was administered to REN cells, in growing conditions. Cells were lysed at 24 hours, 48 hours and 72 hours post-treatment and the degree of eIF6 phosphorylation was analyzed by 2-D electrophoresis, followed by Western Blot analysis. Growing REN cells showed 4 well-focused spots compatible with eIF6 phosphorylation state. Cells treated with 1 μM Enzastaurin, up to 48 hours, showed 3 spots compatible with 1–2 phosphate groups. Long-term treatment (72 hours) of Enzastaurin augmented dephosphorylation of eIF6. Finally, cell lysates treated with phosphatase showed a single focused spot (Figure 2D). Enzastaurin does not affect the stability of both eIF6 and PKCβ, but the latters are more expressed in REN cells compared to non-tumoral Met-5A cells (Supplementary Figure S2). It was recently reported that Enzastaurin affects the phosphorylation of the downstream target of mTORc1 kinase, 4E-BP1, the main mediator of cap-dependent translation [34]. However, in MPM both 4E-BP1 and rpS6 were phosphorylated in the presence of Enzastaurin (Figure 2E). In conclusion, the MPM cell line REN shows eIF6 PKC-dependent hyperphosphorylation and can be used to investigate the effects of eIF6 depletion and dephosphorylation on MPM growth.

### eIF6 antiassociation activity is important for recycling inactive 80S

**Figure 3:**
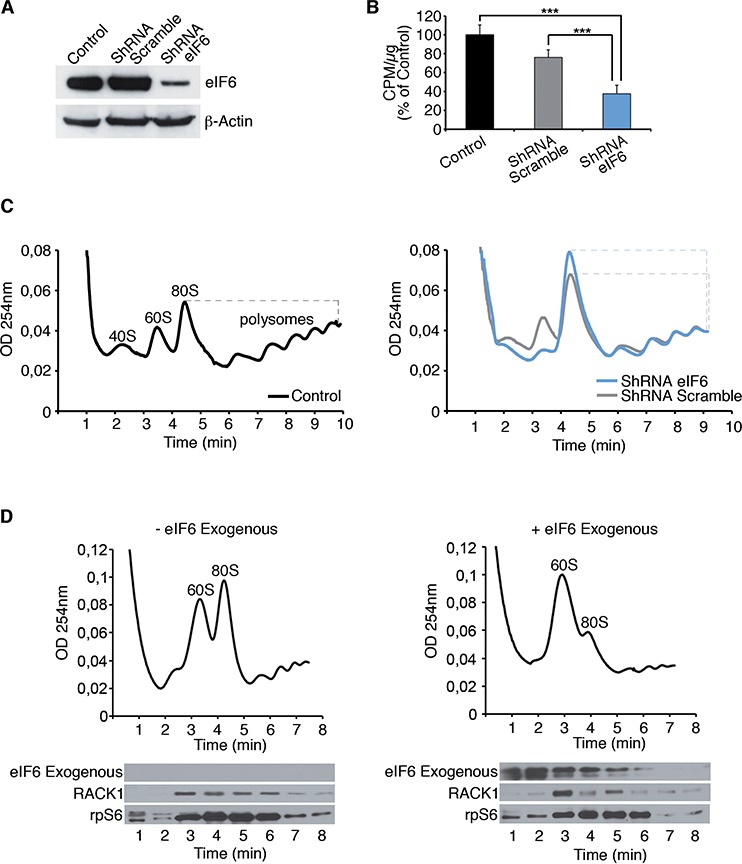
eIF6 antiassociation activity modulates the inititiation of translation of REN cells **A.** Western Blot analysis on REN cells for eIF6 expression in all considered conditions; data are normalized to β-Actin. **B.** Mean of three independent methionine incorporation experiments indicates that eIF6 reduction affects protein synthesis of REN cells. **C.** Polysomal profiles show that partial depletion of eIF6 causes 80S accumulation and reduced translation. 80S/polysomes peaks in each graph are indicated by dashed line. **D.** 5 μg of recombinant biotinylated eIF6 protein is added to polysomes extracts of REN cells: exogenous eIF6 causes the dissociation of inactive 80S, in MPM cell line. Western Blot analysis on recovered fractions derived from polysomal profiles exhibits the distribution of indicated proteins. Experiments are performed 72 hours post-lentiviral transduction. Data, derived from three independent experiments, are represented as mean ± SD. Statistical *p*-values were calculated by two-tailed *t*-test, as above (***p ≤ 0.001).

eIF6 acts in the regulation of translation initiation. We performed a methionine incorporation assay and polysomal profiles on REN cells, to analyze whether eIF6 levels affect initiation (Figure 3). We show a representative experiment performed on REN cells with either normal eIF6 protein levels (control and ShRNA Scramble), or reduced ones (ShRNA eIF6). Western Blot analysis displays eIF6 protein expression (Figure 3A). Experiments were performed 72 hours after lentiviral transduction. In summary eIF6 depletion caused a significant protein synthesis reduction (Figure 3B) and led to a slight decrease of polysomes accompanied by 80S increase, as shown in Figure 3C. Since Enzastaurin modulated eIF6 activity, we performed polysomal profiles and Methionine incorporation on REN cells treated with the drug. We observed that Enzastaurin caused an increase of 80S peak in REN cells but not in Met-5A (Supplementary Figure S3A). Enzastaurin inhibited also methionine incorporation in REN cells (Supplementary Figure S3B).

Translational inhibition by eIF6 depletion, *in vivo*, is in line with the fact that eIF6 is not strictly necessary for translation, but is rate-limiting for oncogene-induced protein synthesis [21]. Next, we analysed in an *ex-vivo* experiment the requirement for eIF6 on 80S cancer ribosomes. We prepared polysomes extracts from REN cells and added recombinant eIF6. Figure 3D shows that eIF6 addition can dissociate inactive 80S, as shown by the drop in the 80S peak and the increase of free 60S. We recovered all fractions derived from polysomal profiles in order to analyze proteins distribution by Western Blotting: exogenous eIF6 was detected on soluble and 60S fractions, consistent with the dissociation data. Taken together our data indicate that eIF6 activity in cancer cells is necessary for keeping ribosomes dissociated, and for initiating new protein synthesis. We asked whether this activity is important for tumor growth.

### eIF6 reduction and dephosphorylation slow cell growth in cultured cells

**Figure 4:**
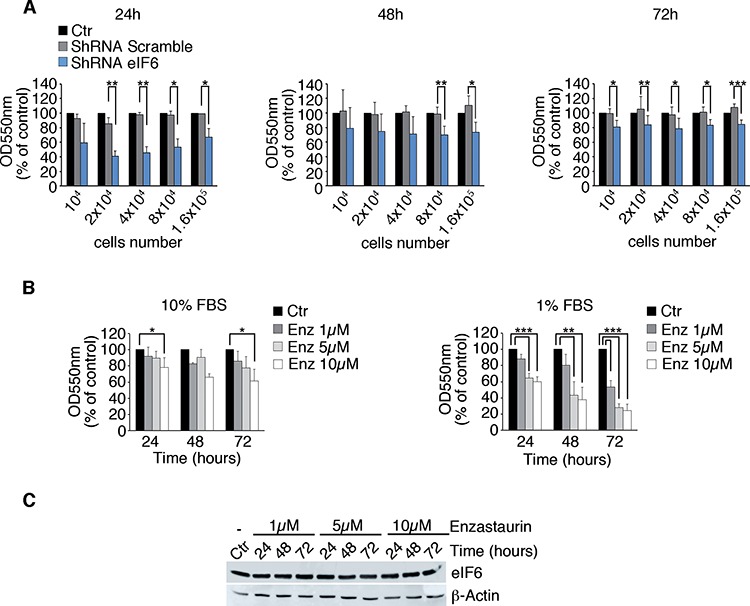
Partial depletion of eIF6 and Enzastaurin treatment affect growth of REN cells **A.** Proliferation rate of REN cells previously infected with the described lentiviruses is analyzed by MTT Assay. Cells with reduced eIF6 protein levels proliferate less at indicated times. **B.** MTT assay on REN cells treated with 1 μM, 5 μM and 10 μM of Enzastaurin: high doses and long-term treatment with Enzastaurin indicate its cytostatic effect, that becomes stronger under serum deprivation. **C.** Western Blot analysis reveals that Enzastaurin does not affect eIF6 protein levels even at high concentrations. All values represent the mean ± SD. Results are representative of three independent experiments. Asterisks indicate a statistically significant change obtained by two-tailed Student *t*-test (*p ≤ 0.05; **p ≤ 0.01; ***p ≤ 0.001).

Established that eIF6 is hyperexpressed and hyperphosphorylated in MPM and in the REN cell line, we asked whether its depletion or dephosphorylation affected growth. We analysed MPM cells growth at 24, 48 and 72 hours after plating and upon eIF6 depletion (Figure 4A, REN cells; Supplementary Figure S4, MM98, sarcomatous, MSTO-211H, biphasic). MTT assay revealed that the proliferation rate of eIF6 depleted cells was slightly reduced compared to the control, *in vitro*. The reduced proliferation was not associated to an increase of the apoptotic rate, in REN cells (Supplementary Figure S5A). In parallel, we performed a MTT Assay on REN cells, treated with 1 μM, 5 μM and 10 μM of Enzastaurin and we measured the proliferation rate at the indicated time points (Figure 4B). Enzastaurin reduced REN growth, indicating its cytostatic effect. The effect was more evident in low-serum conditions. eIF6 protein levels were very similar at all time points upon Enzastaurin treatment at the indicated concentrations (Figure 4C). Furthermore, we performed FACS analysis on synchronous REN cells, with normal or depleted eIF6 protein levels, and/or treated with 1 μM Enzastaurin. Data confirmed that eIF6 reduction impaired G1/S progression and caused a reduced number of cycling cells in G2/M phase. Similar results were obtained with Enzastaurin treatment (Supplementary Figure S5). We also quantitated the apoptotic rate of all these cells after 72 hours of treatment: we found that the percentage of cell death was similar in all considered conditions (Supplementary Figure S5). In conclusion, both shRNA for eIF6 or Enzastaurin treatment slightly reduce proliferation in cultured REN cells, *in vitro*.

### eIF6 depletion and Enzastaurin administration have a protective effect, *in vivo*

**Figure 5:**
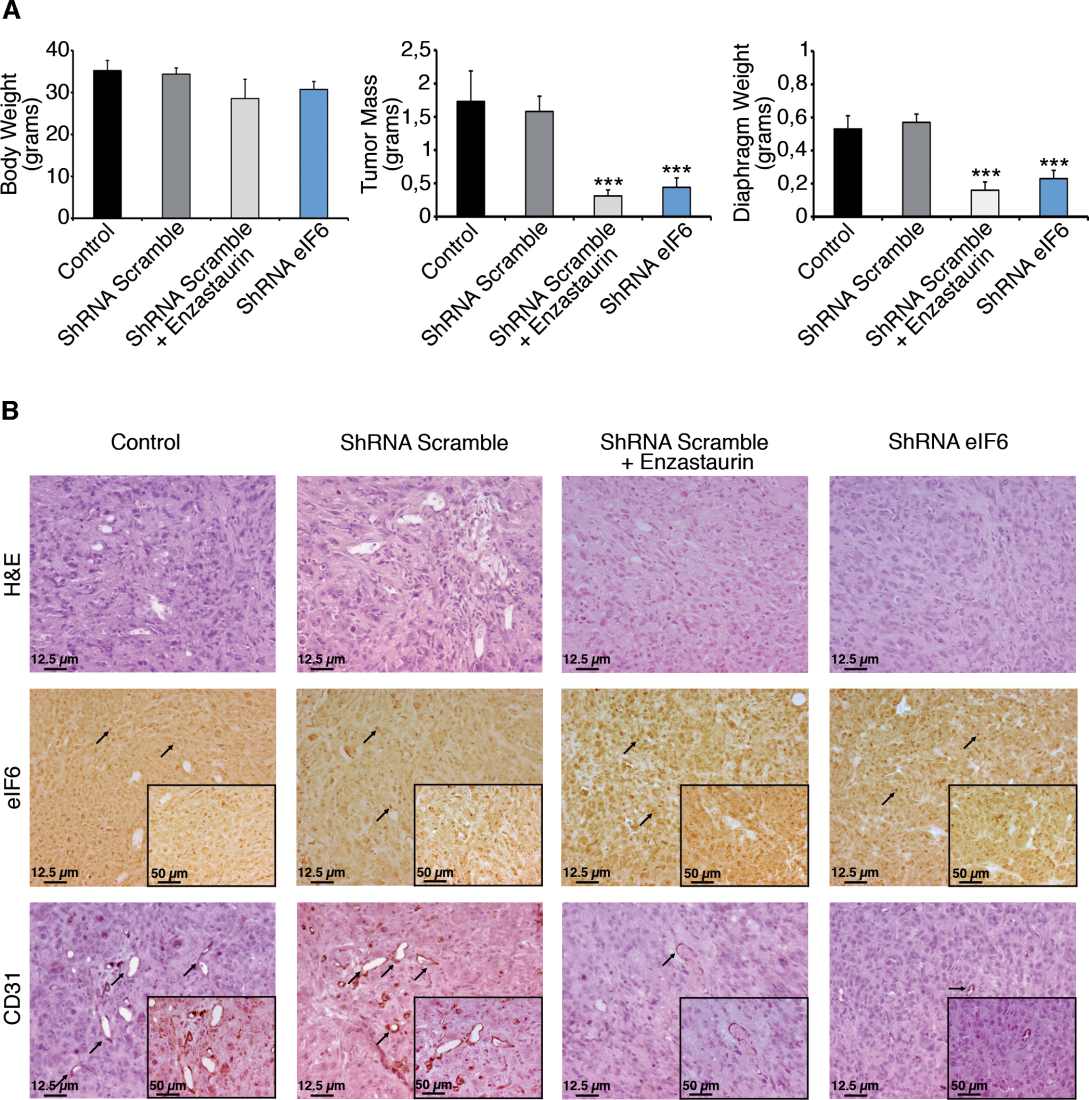
Enzastaurin administration and eIF6 depletion have a protective effect, *in vivo* **A.** REN cells with either wt or depleted eIF6 protein are injected (i.p.) in NOD-SCID mice. Experimental groups are indicated (control mice *n* = 3; mice with ShRNA Scramble plus placebo *n* = 7; mice with ShRNA Scramble plus Enzastaurin *n* = 7; mice with ShRNA eIF6 *n* = 7). A cohort of control mice was treated with Enzastaurin (75 mg/kg) twice daily for 5 weeks. Mice were sacrified two months after tumoral cells injection. eIF6 depletion and Enzastaurin administration reduce tumor masses weight and diaphragm metastasis. **B.** IHC stainings of tumors recovered from NOD-SCID mice. Tissue morphology is evidenced with Hematoxylin and Eosin staining; eIF6 is overexpressed both in the nucleoli (black arrows) and in the cytosol of tumoral cells; staining for CD31 (black arrows) reveals positive vessels; neo-angiogenesis is diminished both in eIF6 depleted conditions and upon drug administration. Scale bar is indicated. Data are represented as mean ± SD. Statistical *p*-values were calculated by two-tailed *t*-test (***p ≤ 0.001).

Then, we addressed the role of eIF6 activity and Enzastaurin, *in vivo*. We developed a murine MPM model by injecting REN cells into immunocompromised NOD-SCID mice. We injected i.p. 10 millions cells/mouse with either wt eIF6 or eIF6 depleted cells. A group of control mice was treated with Enzastaurin: administration (75 mg/Kg) was performed by gavage twice/daily, starting at day 7 after injection and suspending it after 5 weeks. Mice were sacrificed 60 days after cells injection and tumor mass was analyzed. By autopsy, we measured the weight of total body, tumor mass, spleen and diaphragm and we scored for developed metastasis and hemorrhage. Data are summarized in Figure 5A and in Supplementary Table S2: mice injected with REN cells depleted of eIF6 showed reduced tumor mass weight, indicating that the amount of eIF6 is a limiting factor for cellular growth, *in vivo*. These mice also revealed less metastasis, since the diaphragm weight is reduced. Enzastaurin administration provided also a protective effect against tumor growth: indeed, tumor mass was strongly reduced, metastasis were limited to diaphragm and hemorrhage was mild. Both Enzastaurin-treated tumors and shRNA eIF6 tumors recovered from NOD-SCID mice showed less CD31 and VEGFA-positive cells, indicating reduced angiogenesis and close correlation with diminished solid tumor growth and metastasis (Figure 5B, Supplementary Figure S6). These findings may be in agreement with the protective role of eIF6 depletion and/or inactivation by Enzastaurin in neo-angiogenesis and metastasis development [35].

Taken together, these data suggest that either inhibition of eIF6 expression or eIF6 phosphorylation is effective *in vivo*, and that eIF6 is potentially targetable by Enzaustarin.

### eIF6 depletion and Enzastaurin cause metabolic changes of cancer cells

**Figure 6:**
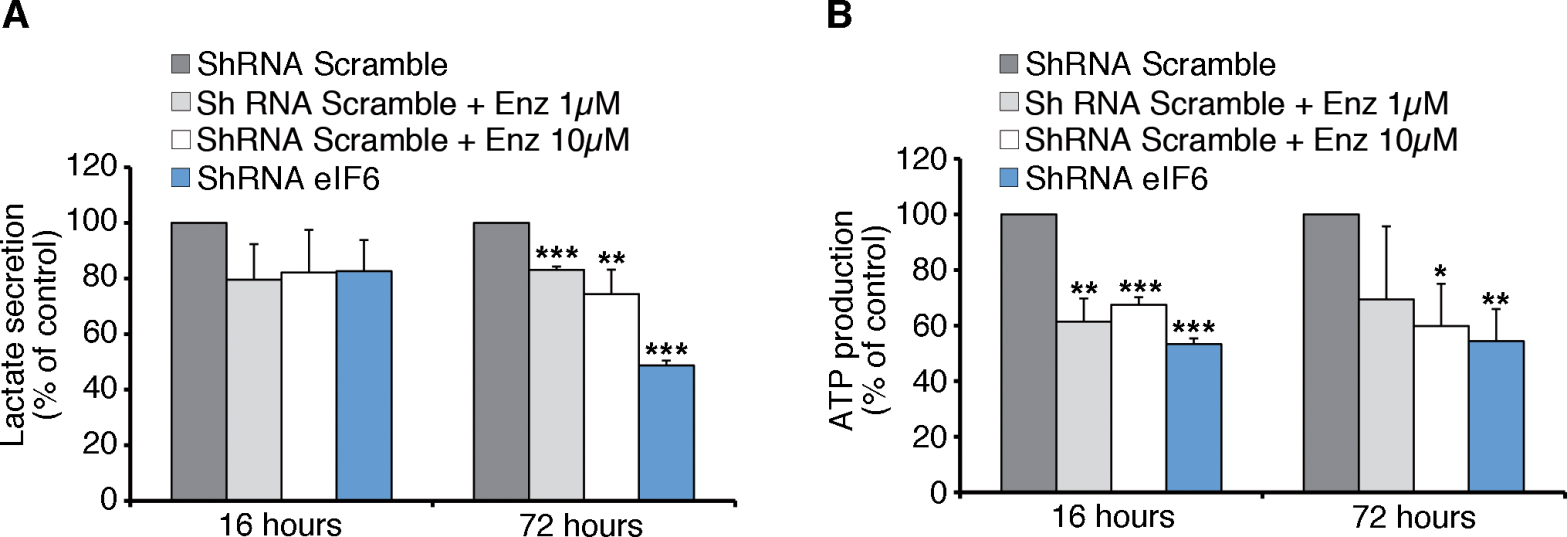
Anti-Warburg effect: eIF6 depletion causes less Glycolysis and ATP content in REN cells **A.** Seventy-two hours after lentiviral infection, REN cells are treated with Enzastaurin for 16 or 72 hours at indicated concentrations. Lactate secretion, an index of glycolytic flux, is significantly reduced in eIF6-depleted and in Enzastaurin-treated cells. **B.** ATP content depends on eIF6 levels and Enzastaurin treatment: acute depletion of eIF6 and Enzastaurin treatment lead to a reduction of ATP levels of REN cells. All values represent the mean ± SD. Results derive from three independent experiments. Asterisks indicate a statistically significant change obtained by Student *t*-test (*p ≤ 0.05; **p ≤ 0.01; ***p ≤ 0.001).

**Figure 7:**
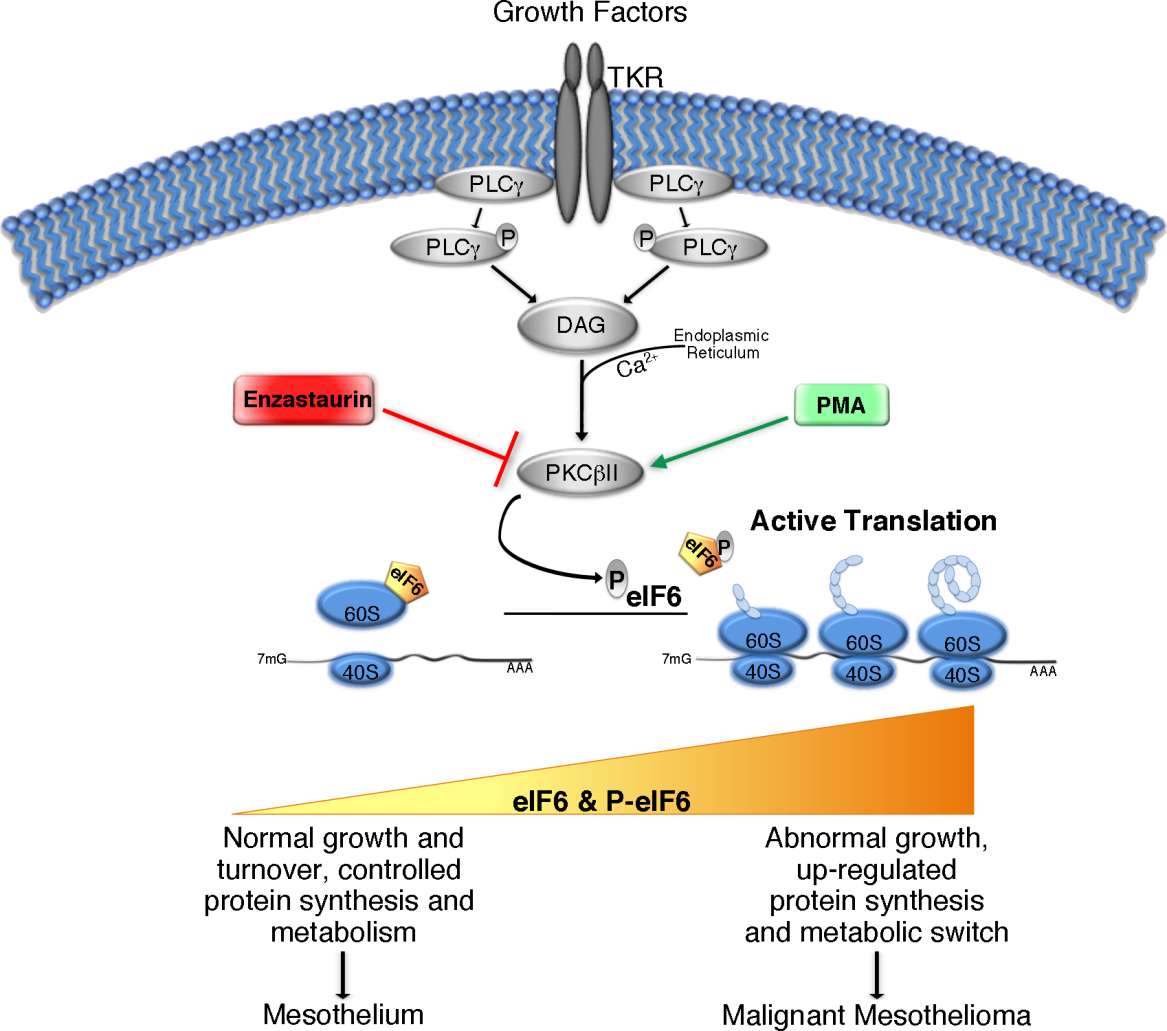
Simplified graphical summary of eIF6 activity in Malignant Pleural Mesothelioma Growth factors stimulation activates PLCγ enzyme, which catalyzes DAG formation, and induces Ca^2+^ release from Endoplasmic Reticulum into the cytoplasm. DAG, in the presence of Calcium, activates PKCβII. The latter phosphorylates eIF6, leading to its release from mature 60S ribosomal subunit. eIF6 activation allows 80S complex assembly and initiation of translation. PMA and Enzastaurin are able to stimulate or inhibit respectively, PKCβ kinase activity. The translational rate increases during tumorigenesis. eIF6 protein levels and its activity could modulate protein synthesis, metabolic status and cell growth: in MPM, contribution of both eIF6 hyperexpression and eIF6 hyperphosphorylation promotes protein synthesis, aerobic glycolysis (metabolic switch) and uncontrolled cellular growth, giving rise to tumor development and malignancy.

The protective role of reduced eIF6 and Enzastaurin administration *in vivo*, compared to the modest effects on cell growth *in vitro* raises the question of whether this effect could be linked to metabolic changes of tumoral REN cells. This selective effect would be consistent with the limited effect of eIF6 depletion on basal translation [20, 36]. A screening for eIF6-regulated genes in the liver, showed eIF6-mediated translation of several transcription factors involved in metabolism, resulting in the regulation of glycolysis [37]. Here, we show that depletion of eIF6 by lentiviral ShRNA on REN cells, and Enzastaurin treatment led to a reduction of lactate secretion, an index of glycolytic flux (Figure 6A). In both cases ATP production was significantly reduced in each considered time (Figure 6B). In summary eIF6 activity is required for a glycolytic switch that may account for its need for tumor growth *in vivo* (Figure 7).

## DISCUSSION

We show that eIF6 is highly expressed and activated in malignant mesotheliomas, and that inhibition of either its activity or phosphorylation reduces tumor burden and tumor growth. Our data further establish the deregulation of the translational machinery in mesothelioma cells, suggesting that this tumor is rather peculiar in its capability to sustain translation, being insensitive to inhibition of the mTOR pathway [30]. We will discuss our findings according three lines, the relevance for malignant mesothelioma, the feasibility and significance to target eIF6, and the molecular mechanism which may account for the increased eIF6 expression in mesothelioma.

Malignant mesothelioma has three main morphological phenotypes, epithelial, sarcomatoid and biphasic. Most tumors arise in the pleura and are epidemiologically linked to asbestos exposure. However, peritoneal mesothelioma also occurs, it is very rare and does not correlate with asbestos exposure [28]. Malignant mesotheliomas originate, by contrast to other tumors, as polyclonal tumors [38]. Genetic analysis of abnormalities has scored an heterogeneous mutational landscape with three predominating lesions, NF2, BAP1 and CDKN2A [39]. miRNA expression is highly variable [40]. Thus, by all means MPM can be defined as a highly heterogeneous cancer. What is not heterogeneous is the (non) response to therapy. MPM is generally found to be resistant to conventional forms of therapy, such as pemetrexed and cisplatinum combination chemotherapy [41]. Thus, on one side, tumor heterogeneity is a major obstacle for applying targeted therapies to mesothelioma and on the other, conventional therapies do not work. Since the components of the translational apparatus integrate different oncogenic pathways, targeting the components of this machinery may overcome tumor heterogeneity. Moreover, malignant cells exhibit an increase of the translational machinery suggesting “addiction” to high protein synthesis [4]. The fact that eIF6 is hyperexpressed and hyperphosphorylated in “MPM” suggests it may be a good target.

There is substantial body of evidence that eIF6 is rate-limiting for cancer cells. First, overexpression of eIF6 is a driver of cancer. Enlargement of eIF6 containing nucleoli is a feature of aggressive colorectal tumors [26]. Soft agar assay of eIF6^+/−^ mouse embryonic fibroblasts transduced with either dominant negative p53 tumor suppressor plus H-rasV12 or with Myc plus H-rasV12 display a 70% reduction in transformed colonies, compared to the eIF6^+/+^ counterparts [20]. The tumorigenic potential of eIF6 is particularly striking in a mouse model of lymphomagenesis, *in vivo*. In this setting, expression of the Myc oncogene under the control of the enhancer of IgH (Eμ-Myc) in the B cell lineage drives a lethal lymphoma, similar to B-cell lymphomas, with a median survival of only 4 months. Eμ-Myc/ eIF6^+/−^ mice have increased survival, up to 1 year and do not show overt negative phenotypes. Even in the p53^−/−^ genotype, where p53 deletion further accelerates lymphomagenesis due to suppression of apoptosis [42], eIF6 depletion delays tumor development. In addition, eIF6 is amplified in breast luminal cancers [27]. The phosphorylation of Ser235 residue on eIF6 is necessary for transformation and cancer development. eIF6 is controlled by the RACK1/PKCβ axis, allowing active 80S complex formation and leading to initiation of translation [23, 25, 43, 44]. PKCB is a pharmacological target in lymphomas [45], and is expressed in mesotheliomas [46]. We previously showed that inhibition of eIF6 phosphorylation by genetic inactivation of Ser235 is a way to block eIF6 activity [21, 23, 32]. Since eIF6 phosphorylation is driven by the PKCβ axis, we decided to inhibit its activity with Enzastaurin (LY317615). Enzastaurin is a FDA approved potent and selective inhibitor of PKCβ; it exerts its antitumor effects both directly, by suppressing tumor cell proliferation and inducing apoptosis, and indirectly, by blocking tumor induced angiogenesis [33, 47–51]. Here we show that reducing eIF6 levels or treating cells with the PKCβ blocker Enzastaurin, restricts MPM cell line growth, *in vitro*, and tumor development and angiogenesis, *in vivo*, in an immunocompromised murine model. Caveats remain: first, in the long run eIF6 inhibition by Enzastaurin may not be effective since eIF6 has multiple phosphorylation sites in its C-terminus [22], yet poorly characterized. Alternative strategies may be therefore required. Second, we have shown that eIF6 activity in cancer is necessary for dissociating inactive 80S subunits. In this context, several point mutations of eIF6 change the efficiency of eIF6 binding to 60S [52]. Recent work from our laboratory has led to the development of a powerful antiassociation assay which may be used for screening inhibitors of eIF6 function, by small compounds chemical libraries. Similar approaches have been successful in targeting other translation factors like eIF4G [53].

Cancer cells alter and reprogram their metabolism to take advantage for growing and developing tumor. This alteration (Warburg phenomenon) consists of an increase in glycolysis that is maintained in conditions of high oxygen tension (“aerobic glycolysis”) and gives rise to enhanced lactate production and glycolytic ATP generation [54]. Moreover, as well as producing more energy, tumor cells increase lipids synthesis to build membranes during oncogenesis [55]. In our lab, we developed a transgenic mouse model where eIF6 heterozygous mice had approximately 50% of reduction of eIF6 protein [20]. In this *in vivo* model, we recently found that eIF6 translational activity directs a lipogenic and glycolytic program through the regulation of enzymes involved in cholesterol and fatty acid synthesis as well as other transcription factors [37]. These data suggested that a similar event might occur in cancer cells, namely eIF6 favors a glycolytic switch. Here, we found that eIF6 depletion and its inactivation through Enzastaurin significantly impair lactate and ATP production in MPM cells. Therefore, our findings may at least partially explain the anti-cancer role of eIF6 inhibition *in vivo*. Our data are in agreement with the observation that mutation of eIF6 Ser235 to Ala greatly reduces cancer growth *in vivo,* more efficiently than *in vitro*. Since the effects of eIF6 depletion on polysomal accumulation are significant, but modest, we expect that specific mRNAs might be regulated by eIF6 activity, at the translational level in REN cells. It will be therefore interesting to apply to mesothelioma tissues novel technologies as ribosome profiling, in order to isolate them [56].

In conclusion, we suggest that modulation of eIF6 levels and activity may lead to a therapeutical avenue in tumor therapy, especially where eIF4E inhibition by rapalogs is not effective, as in malignant mesothelioma [29, 30].

## MATERIALS AND METHODS

### Mice

All experiments involving mice were performed in accordance with italian national regulations. Experimental protocols were reviewed by local Institutional Animal Care and Use Committees (IACUC form sk481). Eight-week old immunocompromised NOD-SCID mice (Charles River Laboratories) were used for detecting tumor growth after intraperitoneal (i.p.) injection of REN cells, as indicated. REN growth, *in vivo*, is modest [57]. Tumor growth was monitored and animals were sacrificed at maximal survival time, settled at 60 days post-injection.

### Cell lines and lentiviral vectors

For this study we used different MPM cell lines: REN cells for Epithelioid subtype, MM98 for Sarcomatous subtype and MSTO-211H for Biphasic subtype. Met-5A (ATCC^®^ CRL-9444™) are immortalized non-tumorigenic mesothelial cells. REN and MM98 cells were grown in DMEM (Lonza), MSTO-211H were grown in RPMI1640 (Lonza) and Met-5A cells in Medium 199 (Life Tech). All media were supplemented with 10% FBS and 1% penicillin, streptomycin, L-glutamine, and all cells were maintained at 37°C and 5% CO_2_. For Western Blotting analysis, normal human primary mesothelial cells were used as Non-Tumoral control. Cells were purchased from Cambridge Bioscience (Cambridge, UK) and maintained according to manufacturer instructions, up to 3 passages. Densitometric analysis was performed by ImageJ software.

MPM cells were stably infected with either one constitutive lentiviral vector carrying scramble ShRNA, used as control, or one carrying eIF6 ShRNA. Lentiviral vectors, pGIPZ Lentiviral ShRNA, were provided by Open Biosystem. Specifically, mature antisense sequences of consitutive shRNA of eIF6 were: 5′-AGCTTCCTACTAGCACCTG-3′ (V3LMM_421640; GIPZ eIF6 shRNA: RMM4532-EG16418). After lentiviral infection, REN cells were selected with puromycin (1 μg/ml) for 48 hours, expanded and treated for with Enzastaurin as specifically described. Similar results are seen using other eIF6 shRNAs as in [37].

### Antibodies and reagents

The following antibodies were used: rabbit polyclonal antibodies against eIF6 [58], rpS6, phospho-rpS6 (Ser240/244), total 4EBP1 (Cell Signaling), P-PKCβII (Cell Signaling); anti-PKCβII (Santa Cruz); rabbit polyclonal anti-VEGFA (Abcam); mouse monoclonal antibodies against β-Actin (Sigma), PKCβ (BD-Bioscience) RACK1 IgM (BD Transduction Laboratories). Biotin was obtained from Pierce, EuroClone (EZ-LINK NHS-LC-BIOTIN). Lambda Protein Phosphatase (Lambda PP) was provided by NEB. Enzastaurin was provided by Eli Lilly and Company (Indianapolis, USA). eIF6 recombinant protein was produced in E. Coli by simultaneous co-expression with chaperones [21], followed by affinity chromatography and size exclusion chromatography (SEC; GE Healthcare).

### mRNA extraction and real-time RT–PCR

Total RNA was extracted with TRIzol reagent (Invitrogen). After treatment of total RNA with RQ1 RNase-free DNase (Promega), reverse transcription was performed with MMLV reverse transcriptase enzyme (Promega) according to the manufacturer's instructions. Reverse transcribed complementary DNA (100 ng) was amplified with the appropriate primers. Taqman probes specific for eIF6 (Hs00158272_m1) and 18S rRNA as an internal standard, were used. Target mRNA quantification by quantitative reverse-transcriptase PCR using ΔΔCt-method using Taqman Universal PCR Master Mix (4304437; Life Technologies) was performed on an ABIPRISM 7900HT Sequence Detection System (Applied Biosystems). Results are represented as means ± SD of at least three independent experiments.

### Cell proliferation, cell cycle and cell death analysis

Proliferation rate of MPM cells was analysed by MTT test: briefly, cells were plated in 96 wells plates at different concentrations, and assayed after 24, 48 and 72 hours. MTT (3-(4, 5-dimethylthiazolyl-2)-2, 5-diphenyltetrazolium bromide) was added and left on cells for 3 hours at 37°C and 5% CO_2_. The resulting intracellular purple formazan was solubilized with SDS and quantified by spectrophotometer at λ = 550/650 nm. Cell cycle analysis was performed on G1 synchronized REN cells. Cells were starved in DMEM without FBS for 12 hours, and then in PBS plus 0,5 mM MgCl2, 10 mM D-Glucose, 1 mM CaCl2 for 3 hours. At the indicated time points, cells were fixed, stained with propidium iodide (PI) and acquired on a BD FACS CANTO II flow cytometer. Cell cycle analysis was performed using the FCS Express software (BD). Cell death detection was performed using APC-Annexin V (BioLegend). Each experiment was done at least in triplicate.

### Polysomal profiles

Growing cells were lysed using a glass douncer in 50 mM Tris HCl pH 7.8, 240 mM KCl, 10 mM MgSO_4_, 5 mM DTT, 250 mM sucrose, 2% Triton X-100, 90 μg/ml cicloheximide, 30 U/ml RNasin. Following clearing, the equivalent of two-hundreds micrograms of RNA was loaded on a 15%–50% sucrose gradient dissolved in 25 mM Tris HCl pH 7.4, 25 mM NaCl, 5 mM MgCl2, 1 mM DTT and spun at 260,000 g for 3 h 30 min with SW41Ti swing rotor (Beckman Coulter). The gradient was then analyzed by continuous flow absorbance at 254 nm, recorded by BioLogic LP software (BioRad). Peaks for 40S, 60S, 80S and polysomes were quantified. For dissociation studies, total extracts of REN cells were incubated 2 minutes at 37°C, with 5 μg of recombinant eIF6 protein or matched controls (PBS; denatured protein), and separated on a 7%–45% sucrose gradient. Extracts containing up to 200 micrograms of RNA were loaded on a 7%–45% (w/v) sucrose gradient containing 50 mM Tris-acetate pH 7.5, 50 mM NH_4_Cl, 12 mM MgCl_2_ and 1 mM DTT, and centrifuged in a Beckman SW41 Ti rotor for 3 h 30 min at 260,000 g. The gradient was analyzed as above. In addition, individual fractions were collected. Fractions were precipitated with 10% trichloroacetic acid (TCA) [23], separated on SDS-PAGE and analyzed by Western blot.

### Datamining

Datasets were retrieved by GEO databases. The affy package was then used to carry out RMA based normalization. Quantitation of target genes was performed by setting expression thresholds at upper one/third. Calculation was performed as follows: original set of microarray data was retrieved from GSE2549. The dataset contains 54 MMP patients. Samples without follow-up survival were discarded, obtaining 42 patients. Expression data on eIF6 and PRKCB were retrieved. Retrieved values ranged for eIF6 from 106 (min) to 468 (max), and for PRKCB from 62 (min) to 403 (max). Assuming that eIF6 hyperphosphorylation and overexpression were linked, we calculated the combined expression by multiplying the eIF6 × PRKCB values. Samples which gave a result in the first quartile of combined expression (practically with values above 1.5 fold the average expression of eIF6 and PRKCB) were compared to the others with the null hypothesis that combined eIF6-PRKCB expression had no effect on survival. Statistical analysis was performed by a paired *t*-test.

### Immunohistochemistry

Immunohistochemical and histological analysis were performed on paraffine-embedded human mesothelioma tissues. Immunoistochemistry (IHC) for eIF6 and calretinin were done using the Vectastain Elite ABC kit (Vector), as previously described [20, 26, 28]. Some sections were counterstained with Hematoxylin-Eosin (H&E).

### Two-dimensional (2-D) gel electrophoresis

Protein extracts of REN, Met-5A and tumor samples, in all described conditions were examined in 2-D gel electrophoresis. Samples were lysed in SDS-free RIPA buffer and proteins were precipitated with 10% TCA. Pellets were resuspended in 2-D buffer (7 M Urea, 2 M Thiourea, 50 mM DTT and 4% CHAPS) and 100 μg of proteins were isoelectrofocused. The first dimension was performed on Ready Strip IPG (pH 3.9–5.1; Biorad). For the reduction/alkylation step, the strips were incubated with re-equlibration buffer (50 mM Tris-HCl, pH 8.8, 6 M urea, 30% glycerol, 2% SDS, bromophenol blue) plus DTT and re-equilibration buffer plus iodoacetamide, respectively. Then, the strips were subjected to SDS/PAGE for the second dimension. Proteins were transferred on PVDF membrane and subsequently incubated with eIF6 monoclonal antibodies [21]. The signal was detected with an anti-mouse secondary antibody and ECL substrate kit (GE Healthcare). Each experimental sample was run at least twice, and at least three different biological replicates were analyzed.

### Measurements of lactate secretion and ATP content

REN cells were plated at 2 × 10^5^ cells/well in 12-well dishes in high-glucose medium for 24 hr [59]. Cells were switched to serum-free high-glucose (4,5 g/L)/high insulin (100 nM) medium for 4 hr. Lactate secreted into the medium was measured using a fluorogenic assay, Lactate Assay Kit (Biovision). Average of fluorescent intensity was calculated for each condition replicates. Values were normalized to protein content obtained from the same wells. For ATP measurements, samples were homogenized in ice-cold ATP buffer (20 mM Tris, pH 7.5, 0.5% Nonidet P-40, 25 mM NaCl, 2.5 mM EDTA) for 5 min. Lysates were centrifuged at 13,000 g for 30 min. Proteins were quantitated by BCA analysis. Luminometric determination of ATP was assayed using the ATP-determination kit (Molecular Probes) according to [60].

### Statistical analysis

Each experiment was repeated at least three times, as biological replicates; means and standard deviations between different experiments were calculated. Statistical *p*-values obtained by Student *t*-test were indicated: three asterisks *** for *p*-values less than 0.001, two asterisks ** for *p*-values less than 0.01 and one asterisk * for *p*-values less than 0.05.

## SUPPLEMENTARY FIGURES AND TABLES



## References

[1] Loreni F, Mancino M, Biffo S (2014). Translation factors and ribosomal proteins control tumor onset and progression: how?. Oncogene.

[2] Silvera D, Formenti SC, Schneider RJ (2011). Translational control in cancer. Nat Rev Cancer.

[3] Bhat M, Robichaud N, Hulea L, Sonenberg N, Pelletier J, Topisirovic I (2015). Targeting the translation machinery in cancer. Nature reviews Drug discovery.

[4] Ruggero D (2013). Translational control in cancer etiology. Cold Spring Harb Perspect Biol.

[5] De Keersmaecker K, Atak ZK, Li N, Vicente C, Patchett S, Girardi T, Gianfelici V, Geerdens E, Clappier E, Porcu M, Lahortiga I, Luca R, Yan J, Hulselmans G, Vranckx H, Vandepoel R (2013). Exome sequencing identifies mutation in CNOT3 and ribosomal genes RPL5 and RPL10 in T-cell acute lymphoblastic leukemia. Nature genetics.

[6] Sonenberg N, Hinnebusch AG (2009). Regulation of translation initiation in eukaryotes: mechanisms and biological targets. Cell.

[7] Mamane Y, Petroulakis E, LeBacquer O, Sonenberg N (2006). mTOR, translation initiation and cancer. Oncogene.

[8] Dowling RJ, Topisirovic I, Alain T, Bidinosti M, Fonseca BD, Petroulakis E, Wang X, Larsson O, Selvaraj A, Liu Y, Kozma SC, Thomas G, Sonenberg N (2010). mTORC1-mediated cell proliferation, but not cell growth, controlled by the 4E-BPs. Science.

[9] Hsieh AC, Costa M, Zollo O, Davis C, Feldman ME, Testa JR, Meyuhas O, Shokat KM, Ruggero D (2010). Genetic dissection of the oncogenic mTOR pathway reveals druggable addiction to translational control via 4EBP-eIF4E. Cancer Cell.

[10] Ruggero D, Montanaro L, Ma L, Xu W, Londei P, Cordon-Cardo C, Pandolfi PP (2004). The translation factor eIF-4E promotes tumor formation and cooperates with c-Myc in lymphomagenesis. Nat Med.

[11] Wendel HG, Silva RL, Malina A, Mills JR, Zhu H, Ueda T, Watanabe-Fukunaga R, Fukunaga R, Teruya-Feldstein J, Pelletier J, Lowe SW (2007). Dissecting eIF4E action in tumorigenesis. Genes Dev.

[12] Alain T, Morita M, Fonseca BD, Yanagiya A, Siddiqui N, Bhat M, Zammit D, Marcus V, Metrakos P, Voyer LA, Gandin V, Liu Y, Topisirovic I, Sonenberg N (2012). eIF4E/4E-BP ratio predicts the efficacy of mTOR targeted therapies. Cancer Res.

[13] Dilling MB, Germain GS, Dudkin L, Jayaraman AL, Zhang X, Harwood FC, Houghton PJ (2002). 4E-binding proteins, the suppressors of eukaryotic initiation factor 4E, are down-regulated in cells with acquired or intrinsic resistance to rapamycin. J Biol Chem.

[14] Mancino MGS, Terragna C, Cavo M, Biffo S (2013). Cap dependent translation contributes to resistance of myeloma cells to bortezomib. Translation.

[15] Li J, Kim SG, Blenis J (2014). Rapamycin: one drug, many effects. Cell Metab.

[16] Choo AY, Yoon SO, Kim SG, Roux PP, Blenis J (2008). Rapamycin differentially inhibits S6Ks and 4E-BP1 to mediate cell-type-specific repression of mRNA translation. Proc Natl Acad Sci U S A.

[17] Di Nicolantonio F, Arena S, Tabernero J, Grosso S, Molinari F, Macarulla T, Russo M, Cancelliere C, Zecchin D, Mazzucchelli L, Sasazuki T, Shirasawa S, Geuna M, Frattini M, Baselga J, Gallicchio M (2010). Deregulation of the PI3K and KRAS signaling pathways in human cancer cells determines their response to everolimus. J Clin Invest.

[18] Valenzuela DM, Chaudhuri A, Maitra U (1982). Eukaryotic ribosomal subunit anti-association activity of calf liver is contained in a single polypeptide chain protein of Mr = 25,500 (eukaryotic initiation factor 6). J Biol Chem.

[19] Sanvito F, Piatti S, Villa A, Bossi M, Lucchini G, Marchisio PC, Biffo S (1999). The beta4 integrin interactor p27(BBP/eIF6) is an essential nuclear matrix protein involved in 60S ribosomal subunit assembly. J Cell Biol.

[20] Gandin V, Miluzio A, Barbieri AM, Beugnet A, Kiyokawa H, Marchisio PC, Biffo S (2008). Eukaryotic initiation factor 6 is rate-limiting in translation, growth and transformation. Nature.

[21] Miluzio A, Beugnet A, Grosso S, Brina D, Mancino M, Campaner S, Amati B, de Marco A, Biffo S (2011). Impairment of cytoplasmic eIF6 activity restricts lymphomagenesis and tumor progression without affecting normal growth. Cancer Cell.

[22] Dephoure N, Zhou C, Villen J, Beausoleil SA, Bakalarski CE, Elledge SJ, Gygi SP (2008). A quantitative atlas of mitotic phosphorylation. Proc Natl Acad Sci U S A.

[23] Ceci M, Gaviraghi C, Gorrini C, Sala LA, Offenhauser N, Marchisio PC, Biffo S (2003). Release of eIF6 (p27BBP) from the 60S subunit allows 80S ribosome assembly. Nature.

[24] Guo J, Wang S, Valerius O, Hall H, Zeng Q, Li JF, Weston DJ, Ellis BE, Chen JG (2011). Involvement of Arabidopsis RACK1 in protein translation and its regulation by abscisic acid. Plant Physiol.

[25] Ruan Y, Sun L, Hao Y, Wang L, Xu J, Zhang W, Xie J, Guo L, Zhou L, Yun X, Zhu H, Shen A, Gu J (2012). Ribosomal RACK1 promotes chemoresistance and growth in human hepatocellular carcinoma. J Clin Invest.

[26] Sanvito F, Vivoli F, Gambini S, Santambrogio G, Catena M, Viale E, Veglia F, Donadini A, Biffo S, Marchisio PC (2000). Expression of a highly conserved protein, p27BBP, during the progression of human colorectal cancer. Cancer Res.

[27] Gatza ML, Silva GO, Parker JS, Fan C, Perou CM (2014). An integrated genomics approach identifies drivers of proliferation in luminal-subtype human breast cancer. Nat Genet.

[28] Carbone M, Ly BH, Dodson RF, Pagano I, Morris PT, Dogan UA, Gazdar AF, Pass HI, Yang H (2012). Malignant mesothelioma: facts, myths, and hypotheses. Journal of cellular physiology.

[29] Grosso S, Pesce E, Brina D, Beugnet A, Loreni F, Biffo S (2011). Sensitivity of global translation to mTOR inhibition in REN cells depends on the equilibrium between eIF4E and 4E-BP1. PLoS One.

[30] Ou SH, Moon J, Garland LL, Mack PC, Testa JR, Tsao AS, Wozniak AJ, Gandara DR (2015). SWOG S0722: Phase II Study of mTOR Inhibitor Everolimus (RAD001) in Advanced Malignant Pleural Mesothelioma (MPM). Journal of thoracic oncology : official publication of the International Association for the Study of Lung Cancer.

[31] Gordon GJ, Rockwell GN, Jensen RV, Rheinwald JG, Glickman JN, Aronson JP, Pottorf BJ, Nitz MD, Richards WG, Sugarbaker DJ, Bueno R (2005). Identification of novel candidate oncogenes and tumor suppressors in malignant pleural mesothelioma using large-scale transcriptional profiling. Am J Pathol.

[32] Grosso S, Volta V, Sala LA, Vietri M, Marchisio PC, Ron D, Biffo S (2008). PKCbetaII modulates translation independently from mTOR and through RACK1. Biochem J.

[33] Schwartzberg L, Hermann R, Flinn I, Flora D, Hsi ED, Hamid O, Shi P, Lin BK, Myrand SP, Nguyen TS, Dreyling M (2014). Open-label, single-arm, phase II study of enzastaurin in patients with follicular lymphoma. Br J Haematol.

[34] Dumstorf CA, Konicek BW, McNulty AM, Parsons SH, Furic L, Sonenberg N, Graff JR (2010). Modulation of 4E-BP1 function as a critical determinant of enzastaurin-induced apoptosis. Mol Cancer Ther.

[35] Graff JR, McNulty AM, Hanna KR, Konicek BW, Lynch RL, Bailey SN, Banks C, Capen A, Goode R, Lewis JE, Sams L, Huss KL, Campbell RM, Iversen PW, Neubauer BL, Brown TJ (2005). The protein kinase Cbeta-selective inhibitor, Enzastaurin (LY317615.HCl), suppresses signaling through the AKT pathway, induces apoptosis, and suppresses growth of human colon cancer and glioblastoma xenografts. Cancer Res.

[36] Loreni F, Mancino M, Biffo S (2014). Translation factors and ribosomal proteins control tumor onset and progression: how?. Oncogene.

[37] Brina D, Miluzio A, Ricciardi S, Clarke K, Davidsen P, Viero G, Tebaldi T, Offenhäuser N, Rozmann J, Rathkolb B, Neschen S, Klingenspor M, Wolf E, Gailus-Durner V, Fuchs H, Hrabe de Angelis M (2015). eIF6 coordinates insulin sensitivity and lipid metabolism by coupling translation to transcription. Nature Communications.

[38] Comertpay S, Pastorino S, Tanji M, Mezzapelle R, Strianese O, Napolitano A, Baumann F, Weigel T, Friedberg J, Sugarbaker P, Krausz T, Wang E, Powers A, Gaudino G, Kanodia S, Pass HI (2014). Evaluation of clonal origin of malignant mesothelioma. Journal of translational medicine.

[39] Sekido Y (2013). Molecular pathogenesis of malignant mesothelioma. Carcinogenesis.

[40] Truini A, Coco S, Alama A, Genova C, Sini C, Dal Bello MG, Barletta G, Rijavec E, Burrafato G, Boccardo F, Grossi F (2014). Role of microRNAs in malignant mesothelioma. Cell Mol Life Sci.

[41] Belli C, Fennell D, Giovannini M, Gaudino G, Mutti L (2009). Malignant pleural mesothelioma: current treatments and emerging drugs. Expert opinion on emerging drugs.

[42] Post SM, Quintas-Cardama A, Terzian T, Smith C, Eischen CM, Lozano G (2010). p53-dependent senescence delays Emu-myc-induced B-cell lymphomagenesis. Oncogene.

[43] Brina D, Grosso S, Miluzio A, Biffo S (2011). Translational control by 80S formation and 60S availability: The central role of eIF6, a rate limiting factor in cell cycle progression and tumorigenesis. Cell Cycle.

[44] Guo J, Jin Z, Yang X, Li JF, Chen JG (2011). Eukaryotic initiation factor 6, an evolutionarily conserved regulator of ribosome biogenesis and protein translation. Plant Signal Behav.

[45] Ma S, Rosen ST (2007). Enzastaurin. Curr Opin Oncol.

[46] Faoro L, Loganathan S, Westerhoff M, Modi R, Husain AN, Tretiakova M, Seiwert T, Kindler HL, Vokes EE, Salgia R (2008). Protein kinase C beta in malignant pleural mesothelioma. Anticancer Drugs.

[47] Yang Y, Chen Y, Saha MN, Chen J, Evans K, Qiu L, Reece D, Chen GA, Chang H (2015). Targeting phospho-MARCKS overcomes drug-resistance and induces antitumor activity in preclinical models of multiple myeloma. Leukemia.

[48] Cosenza M, Civallero M, Pozzi S, Marcheselli L, Bari A, Sacchi S (2014). The combination of bortezomib with enzastaurin or lenalidomide enhances cytotoxicity in follicular and mantle cell lymphoma cell lines. Hematological oncology.

[49] Wei XW, Zhang ZR, Wei YQ (2013). Anti-angiogenic drugs currently in Phase II clinical trials for gynecological cancer treatment. Expert Opin Investig Drugs.

[50] Querfeld C, Kuzel TM, Kim YH, Porcu P, Duvic M, Musiek A, Rook AH, Mark LA, Pinter-Brown L, Hamid O, Lin B, Bian Y, Boye M, Day JM, Rosen ST (2011). Multicenter phase II trial of enzastaurin in patients with relapsed or refractory advanced cutaneous T-cell lymphoma. Leuk Lymphoma.

[51] Bodo J, Sedlak J, Maciejewski JP, Almasan A, Hsi ED (2011). HDAC inhibitors potentiate the apoptotic effect of enzastaurin in lymphoma cells. Apoptosis : an international journal on programmed cell death.

[52] Menne TF, Goyenechea B, Sanchez-Puig N, Wong CC, Tonkin LM, Ancliff PJ, Brost RL, Costanzo M, Boone C, Warren AJ (2007). The Shwachman-Bodian-Diamond syndrome protein mediates translational activation of ribosomes in yeast. Nat Genet.

[53] Moerke NJ, Aktas H, Chen H, Cantel S, Reibarkh MY, Fahmy A, Gross JD, Degterev A, Yuan J, Chorev M, Halperin JA, Wagner G (2007). Small-molecule inhibition of the interaction between the translation initiation factors eIF4E and eIF4G. Cell.

[54] Warburg O (1956). On respiratory impairment in cancer cells. Science.

[55] Menendez JA, Lupu R (2007). Fatty acid synthase and the lipogenic phenotype in cancer pathogenesis. Nat Rev Cancer.

[56] Ingolia NT, Brar GA, Rouskin S, McGeachy AM, Weissman JS (2012). The ribosome profiling strategy for monitoring translation *in vivo* by deep sequencing of ribosome-protected mRNA fragments. Nat Protoc.

[57] Volta V, Ranzato E, Martinotti S, Gallo S, Russo MV, Mutti L, Biffo S, Burlando B (2013). Preclinical demonstration of synergistic Active Nutrients/Drug (AND) combination as a potential treatment for malignant pleural mesothelioma. PLoS One.

[58] Biffo S, Sanvito F, Costa S, Preve L, Pignatelli R, Spinardi L, Marchisio PC (1997). Isolation of a novel beta4 integrin-binding protein (p27(BBP)) highly expressed in epithelial cells. J Biol Chem.

[59] Duvel K, Yecies JL, Menon S, Raman P, Lipovsky AI, Souza AL, Triantafellow E, Ma Q, Gorski R, Cleaver S, Vander Heiden MG, MacKeigan JP, Finan PM, Clish CB, Murphy LO, Manning BD (2010). Activation of a metabolic gene regulatory network downstream of mTOR complex 1. Mol Cell.

[60] Lopez-Lluch G, Hunt N, Jones B, Zhu M, Jamieson H, Hilmer S, Cascajo MV, Allard J, Ingram DK, Navas P, de Cabo R (2006). Calorie restriction induces mitochondrial biogenesis and bioenergetic efficiency. Proceedings of the National Academy of Sciences of the United States of America.

