# Supplementary material for: Expression and activity of eIF6 trigger Malignant Pleural Mesothelioma growth in vivo
Source: Oncotarget. 2015 Oct 6;6(35):37471–85. doi: 10.18632/oncotarget.5462 (PMC4741942; doi:10.18632/oncotarget.5462)
Supplement: Supplementary file 1 [file oncotarget-06-37471-s001.pdf]

SUPPLEMENTARY FIGURES AND TABLES

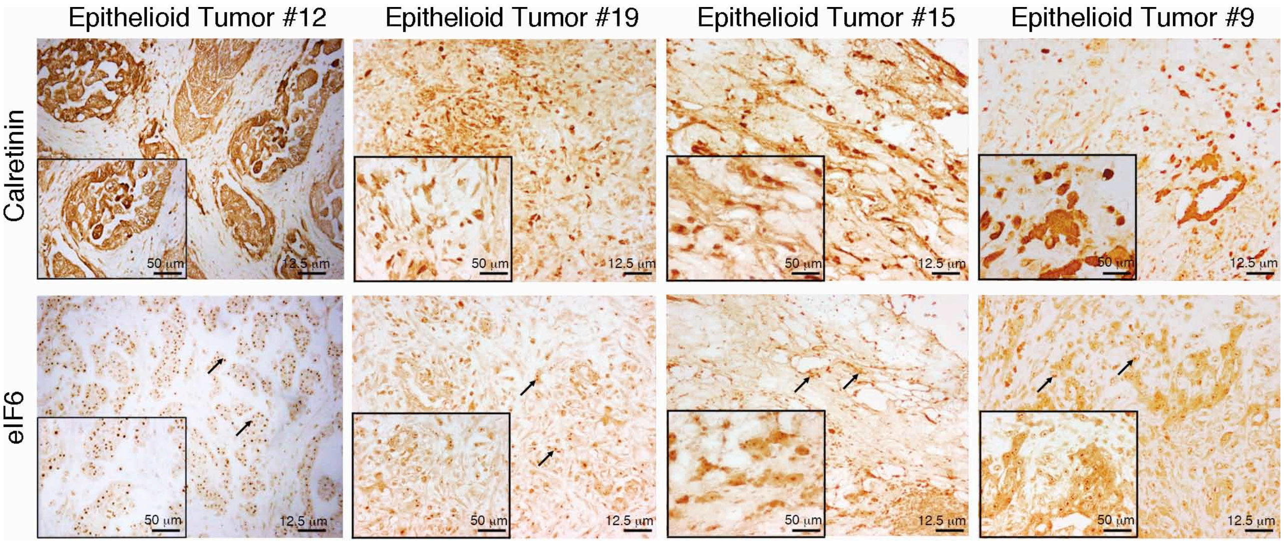

Supplementary Figure S1 (Related to Figure 1): IHC stainings on 4 biopsies of epithelial Malignant Pleural Mesothelioma. Black arrows indicate eIF6 expression in the nucleoli. Calretinin is used as marker of MPM. Scale bar is indicated.

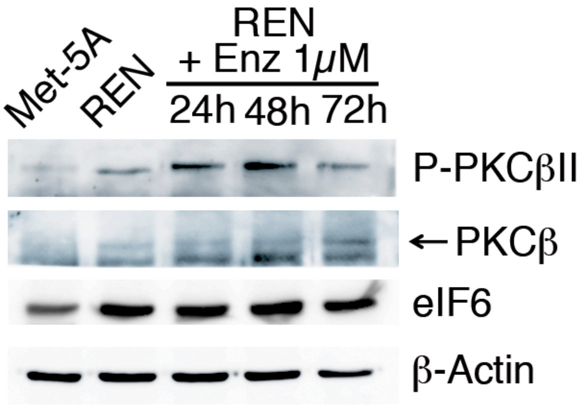

Supplementary Figure S2 (Related to Figure 2): Enzastaurin treatment does not affect eIF6 and PKCβ proteins stability. Western Blot analysis shows that eIF6 and PKCβ are overexpressed in REN cells compared to non tumoral Met-5A cells. Proteins levels are similar upon Enzastaurin treatment in all considered times. Data are normalized to β-Actin. Data are representative of three independent experiments.

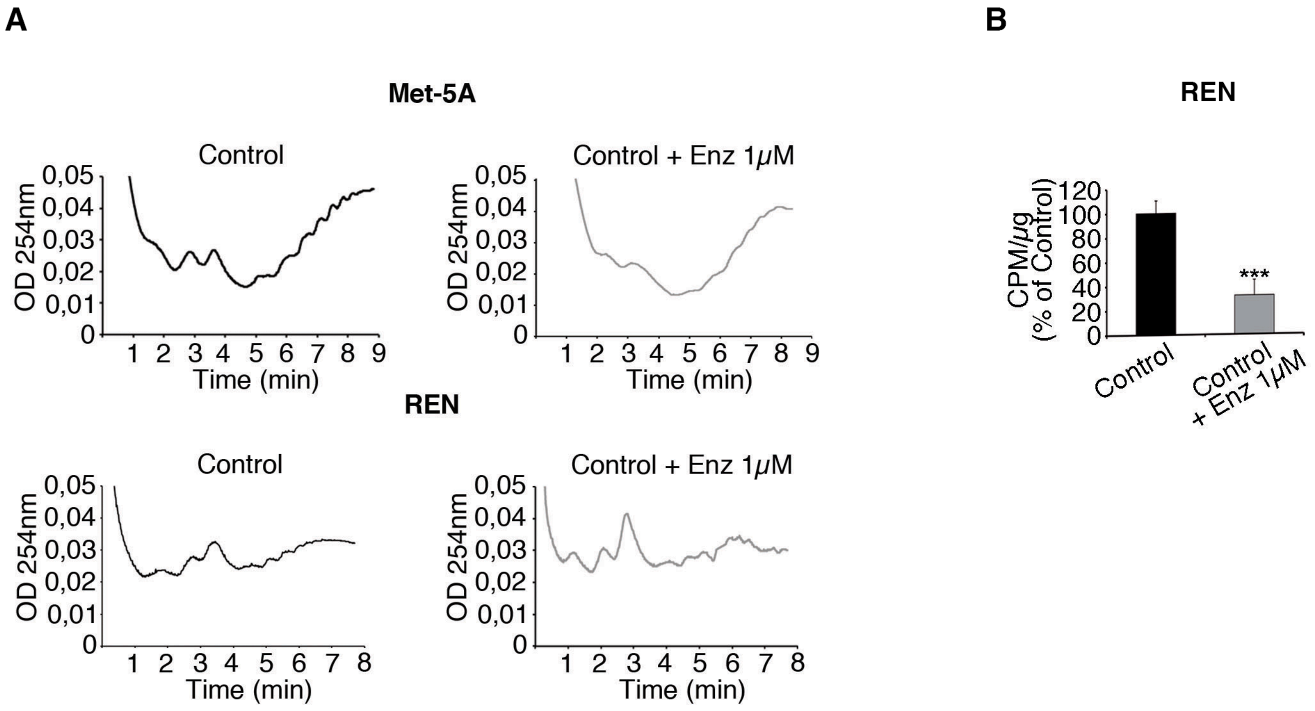

Supplementary Figure S3 (Related to Figure 3): Enzastaurin administration affects protein synthesis in REN cells. A. Representative polysomal profile on REN and Met-5A cells shows that Enzastaurin causes 80S accumulation and reduced translation. **B.** Protein synthesis is significantly impaired upon Enzastaurin treatment in REN cells. All values represent the mean ± SD. Results are representative of three independent experiments. Asterisks indicate a statistically significant change obtained by two-tailed *t*-test (***$p \leq 0.001$).

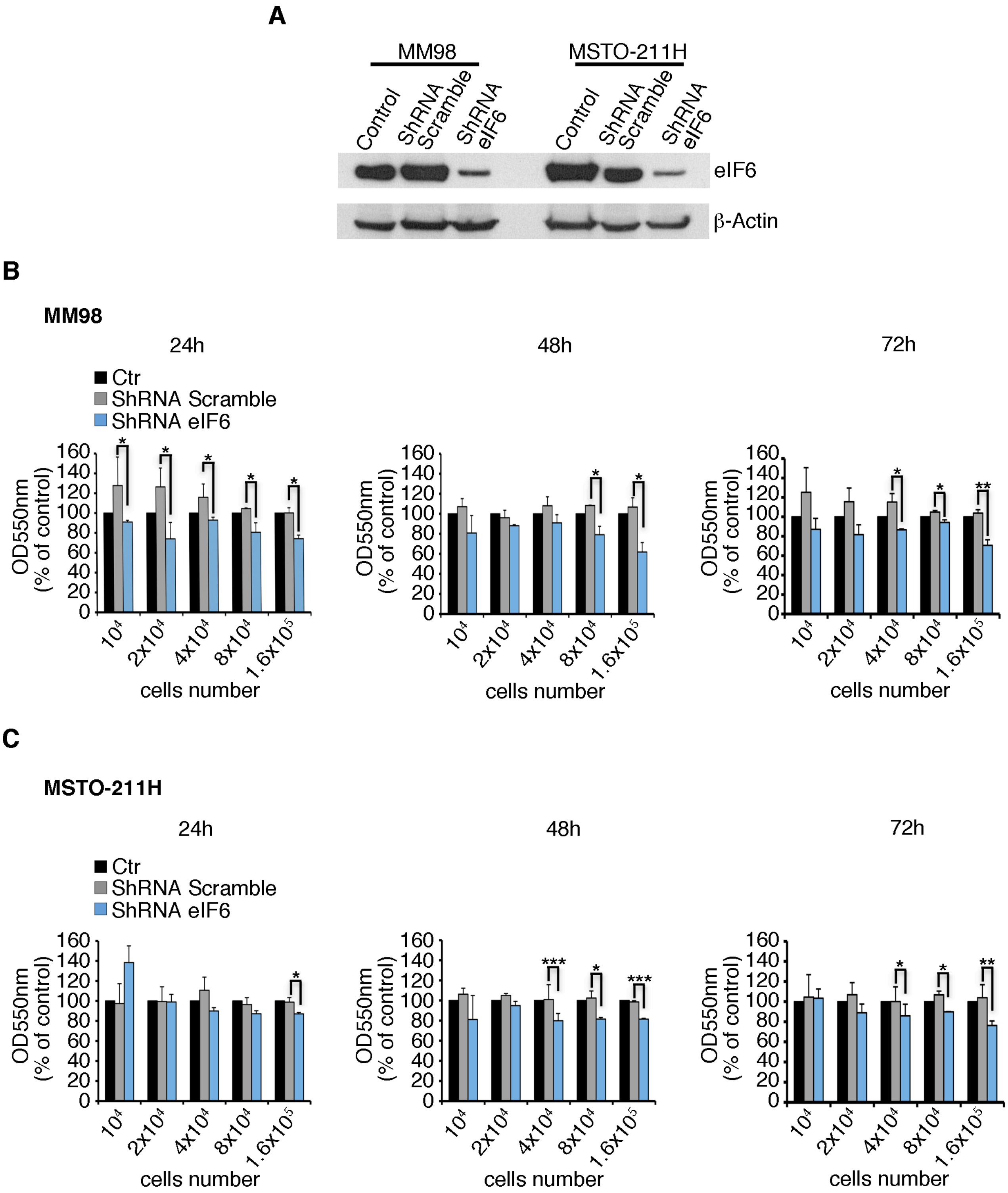

Supplementary Figure S4 (Related to Figure 4): Partial depletion of eIF6 affects proliferation of MPM cell lines. A. Representative Western Blot indicates that shRNA eIF6 transduction reduces eIF6 protein levels in MM98 and MSTO-211H cells. Data are normalized to β-Actin. **B, C.** MTT assay on MM98 and MSTO-211H cells: partial depletion of eIF6 affects MPM cells proliferation at indicated conditions. Data derived from three independent experiments are represented as mean ± SD. Statistical *p*-values were calculated by two-tailed *t*-test ($*p \leq 0.05$; $**p \leq 0.01$; $***p \leq 0.001$).

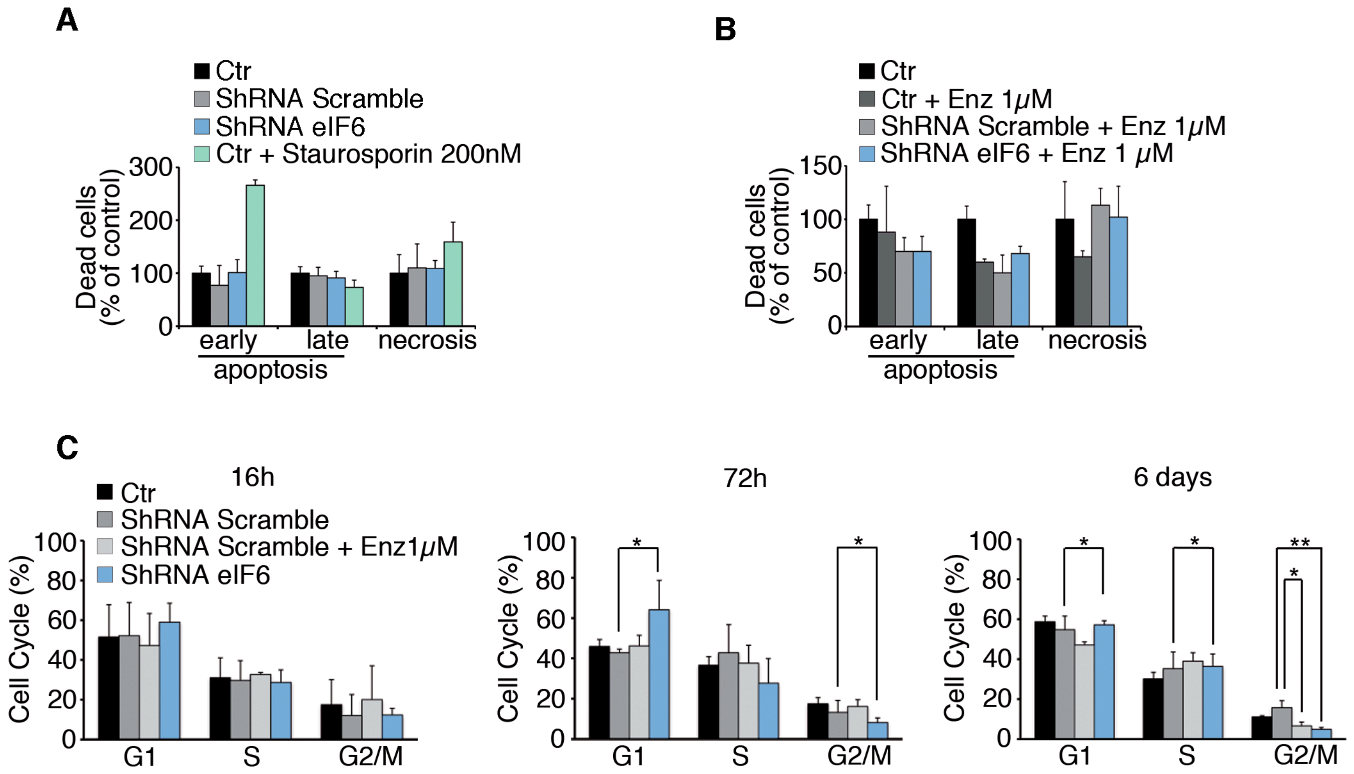

Supplementary Figure S5 (Related to Figure 4): Partial depletion of eIF6 and Enzastaurin treatment cause less growth, but not high apoptosis in REN cells. A, B. FACS analysis show that the apoptotic rate is similar in all considered conditions. **C.** Cell cycle analysis of synchronous REN cells, with normal or depleted eIF6 protein levels, and treated with 1 μM Enzastaurin. eIF6 reduction impairs G1/S progression in synchronised cells and causes a reduced number of cycling cells in G2/M phase, at considered time points. Similar results are obtained with Enzastaurin treatment. All values represent the mean ± SD. Results are representative of three independent experiments. *p*-value, obtained by Student *t*-test, are indicated (*$p \leq 0.05$; **$p \leq 0.01$).

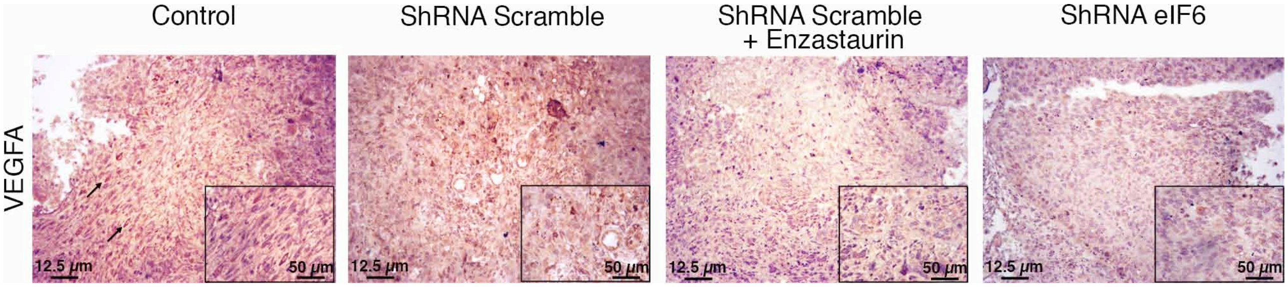

Supplementary Figure S6 (Related to Figure 5): eIF6 depletion and Enzastaurin have an antiangiogenic effect *in vivo*. IHC staining against VEGFA on tumors recovered from NOD-SCID mice displays less VEGFA expression in eIF6 depleted conditions and upon drug administration. Scale bar is indicated.

Supplementary Table S1 Related to Figure 1:

A. List of Human Malignant Pleura Mesothelioma Cases used for Immunohystochemistry analysis. Hospital Dall'Angelo, Pathology, Venice, Italy.

Patients Number/Sex	Age (years)	Therapy	Survival (months)	Histotype
1. Male	55	PI+CT+RT	11	EP
2. Male	58	PPE+CT+RT	9	EP
3. Male	59	TP+CT+RT	42	EP
4. Male	60	PT	15	EP
5. Male	60	CT+RT	42	EP
6. Male	63	TP+CT+RT	38	EP
7. Male	64	PPE	3	S
8. Male	65	PPE+CT	13	EP
9. Male	67	PPE+CT	9	EP
10. Male	68	PPE+CT	10	EP
11. Male	68	PPE+CT	9	EP
12. Male	69	PI+CT+RT	16	EP
13. Male	70	TP+CT+RT	38	EP
14. Male	71	TP+CT+RT	25	B
15. Male	71	TP+CT	17	EP
16. Male	71	TP+CT+RT	25	EP
17. Male	72	PPE	8	EP
18. Male	75	TP+CT	4	EP
19. Male	76	CT+RT	13	EP
20. Male	76	TP+CT	14	EP
21. Male	80	NO	3	S
22. Female	68	CT	6	S
23. Female	75	CT+RT	8	EP
24. Female	77	TP+CT	26	EP\

Histotypes: EP: Epithelioid, B: Biphasic, S: Sarcomatous
Therapy: TP: Total Pleurectomy, PPE: Pleuropneumonectomy, CT: Chemotherapy, RT: Radiotherapy

B. List of Human Malignant Pleura Mesothelioma Cases used for Western Blotting and 2-D analysis. Glenfield Hospital, Leicester, UK.

Patients Number/Sex	Age (years)	Histotype
A. Male	77	Epithelioid
B. Female	62	Epithelioid
C. Male	66	Epithelioid
D. Male	77	Epithelioid
E. Male	59	Epithelioid
F. Male	78	Epithelioid
G. Male	56	Epithelioid
H. Male	61	Epithelioid

C. List of Human Non-Malignant Mesothelioma Pleural Biopsies, used for Immunohystochemistry analysis. Hospital Dall'Angelo, Pathology, Venice, Italy.

Patients Number/Sex	Age (years)	Disease
1. Male	75	Relapsed Pleurisy
2. Female	50	Relapsed Pleurisy
3. Male	71	Lung Carcinoma

Supplementary Table S2 (Related to Figure 5): Autopsies on NOD-SCID mice 60 days after REN cells injection. The number of autopsies is indicated for each group.

Group	Weight (grams)				Metastasis	Intraperitoneal Hemorrhage
	Body	Tumor Mass	Spleen	Diaphragm		
**Control** (*n* = 3)	35,24 ± 2,4	1,73 ± 0,46	0,29 ± 0,045	0,53 ± 0,08	Liver, Diaphragm	Massive
**ShRNA Scramble + placebo** (*n* = 7)	34,37 ± 1,5	1,58 ± 0,23	0,42 ± 0,09	0,57 ± 0,05	Liver, Diaphragm	Massive
**ShRNA Scramble + Enz.** (*n* = 7)	28,56 ± 4,6	0,31 ± 0,09	0,12 ± 0,08	0,16 ± 0,05	Diaphragm	Mild
**ShRNAeIF6** (*n* = 7)	30,73 ± 1,9	0,44 ± 0,14	0,25 ± 0,05	0, 23 ± 0,05	Diaphragm	Massive
